# Supplementary figures and images for: A new, three-dimensional geometric morphometric approach to assess egg shape
Source: PeerJ. 2018 Jun 27;6:e5052. doi: 10.7717/peerj.5052 (PMC6026453; doi:10.7717/peerj.5052)

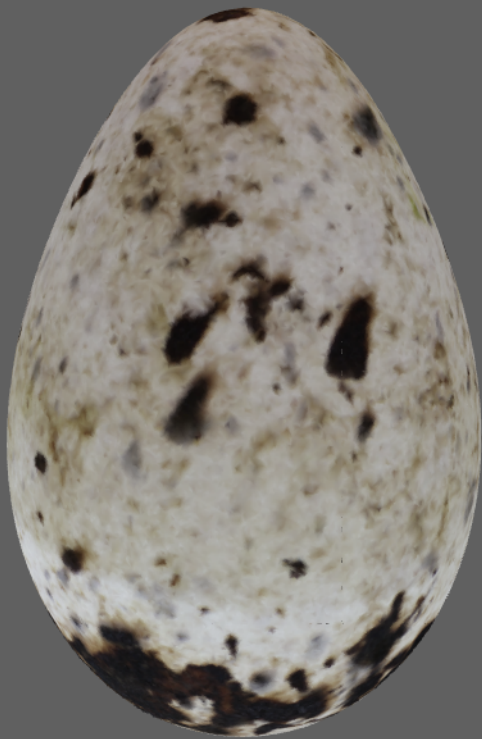

Supplement: Supplemental Information 1 — A total of 145 photographs were taken at multiple angles around the egg. The photographs were processed in Agisoft PhotoScan (v. 1.2.6) to create the 3D model. The specimen (ID number: 2016.R15.Top.LWB) was provided by the Alfred Denny Museum, Sheffield. [file peerj-06-5052-s001.pdf]
