# Supplementary material for: A new, three-dimensional geometric morphometric approach to assess egg shape
Source: PeerJ. 2018 Jun 27;6:e5052. doi: 10.7717/peerj.5052 (PMC6026453; doi:10.7717/peerj.5052)
Supplement: Supplemental Information 2 — Incubation period is from completion to of clutch to hatching. Sources are Higgins, Peter & Steele, 2001; Higgins & Peter, 2002; Higgins, Peter & Cowling, 2006. [file peerj-06-5052-s002.docx]

| Common name | Family | Clade | Population description | Body length range (mm) | Body mass average (mm) | Primary diet | Clutch size average | Clutch size range | Nest material | Nest shape | Nest site | Incubation | Incubation period (days) | Young development |
| --- | --- | --- | --- | --- | --- | --- | --- | --- | --- | --- | --- | --- | --- | --- |
| Grey Shrike-thrush | Pachycephalidae | Whistlers, Allies | Australian | 22-27 | 67.5 | Vertebrates, invertebrates | 3 | 1-4 | Strips of bark | Cup | Hollow | Biparental | 16.9 | Altricial |
| Red-browed Finch | Estrildidae | Waxbills, Allies | Endemic | 10-13 | 9.5 | Small seeds and grasses | 5 | 2-8 | Dry or green grass | Dome or bottle-shaped with tunnel or spout entrance. | In fork among dense foilage | Biparental | 14.6 | Altricial |
| Spiny-cheeked Honeyeater | Meliphagidae | Honeyeaters | Endemic | 22-27 | 44.7 | Fruit, nectar, insects | 2.2 | 1-3 | Grass, spider web | Cup | Live tree or shrub | Unknown | 14.5 | Altricial |
| Superb Fairy-wren | Maluridae | Australasian Wrens | Endemic | 11-14 | 10.6 | Invertebrates | 3.2 | 1-5 | Grass, strips of bark, twigs, moss, fern fronds, spider web/egg-sacs | Dome with side entrance | Live plant | Female | 14 | Altricial |
